# Paraoxonase 1 gene polymorphisms in lipid oxidation and atherosclerosis development

**DOI:** 10.3389/fgene.2022.966413

**Published:** 2022-09-02

**Authors:** Marija Vavlukis, Ana Vavlukis, Katerina Krsteva, Sonja Topuzovska

**Affiliations:** ^1^ University Clinic for Cardiology, Skopje, North Macedonia; ^2^ Faculty of Medicine, Ss. Cyril and Methodius University in Skopje, Skopje, North Macedonia; ^3^ Faculty of Pharmacy, Ss. Cyril and Methodius University in Skopje, Skopje, North Macedonia; ^4^ Institute of Medical and Experimental Biochemistry, Faculty of Medicine, Ss. Cyril and Methodius University in Skopje, Skopje, North Macedonia

**Keywords:** paraoxonase 1, polymorphism, enzyme activity, HDL cholesterol, atherosclerosis

## Abstract

Paraoxonase 1 (PON1) is calcium-dependent aryldialkylphosphatase, thought to possess; anti-oxidant, anti-adhesion, anti-inflammatory, anti-thrombosis and anti-apoptosis effects, as well as lipid-modifying properties. Numerous clinical studies have shown associations between different PON1 polymorphisms and different cardiovascular pathologies. The rs622 (c.575A > G) and the rs854560 (c.163A > T) are the most studied PON1 SNPs in the coding region, with rs705381 (− 162A/G), rs854572 (− 909G/C) and rs705379 (− 108C/T) being the most studied SNPs in the regulatory PON1 gene region. The three major PON1 activities are aryldialkylphosphatase, arylesterase and lactonase activity. The different SNPs affect PON1 serum concentrations and enzyme activity, thus leading to pro-/anti-atherogenic effects. In that setting, it is very difficult to establish as to whether the genotype or phenotype of PON1 is primarily associated with cardiovascular risk. Given the current scientific evidence, PON1 genotyping might be reasonable in patients with high and very high cardiovascular risk.

## Introduction

Paraoxonase 1 (PON1) is a 43–45 kDa glycoprotein aryldialkylphosphatase with 354–355 amino acids. PON1 is thought to prevent atherosclerosis, mainly due to its anti-oxidant activity. It also possesses beneficial effects regarding adhesion, inflammation, apoptosis, thrombosis, as well as lipid-modifying actions (Chistiakov et al., 2017). PON1 stimulates reverse cholesterol transport, reduction of oxidized lipids in both low-density lipoprotein (LDL) and high-density lipoprotein (HDL), inhibits peroxidation of lipoproteins, and maintains the anti-oxidative potential of HDL (Grzegorzewska et al., 2021).

The PON1 gene is located on the 7q21.3 chromosome. PON1 is encoded by a nine exons primary transcript, using classic splice acceptor and donor sites. One of the most studied PON1 nucleotide change is the rs622 (c.575A > G) missense mutation, which leads to a glutamine - arginine replacement at position 192 (p.Gln192Arg). This mutation leads to increased paraoxonase activity (Sikora et al., 2020). rs854560 (c.163A > T) is a missense mutation that results in the replacement of leucine with methionine at position 55 (p.Leu55Met). The c.163T transcript is more labile than the wild-type, resulting in reduction of the PON1 concentration and activity. As opposed to the polymorphisms in the coding region, mutations of the regulatory region, such as rs705379 (− 108C/T), rs854572 (− 909G/C) and rs705381 (− 162A/G), seem to increase serum PON1 levels (Grzegorzewska et al., 2021). PON 1 is thought to have three major activities - aryldialkylphosphatase, arylesterase and lactonase. Even though PON1 exhibits its enzymatic activity on oxidized lipids, the exact physiological substrates for PON1 are poorly understood (Petrič et al., 2021). Of the three main activities, paraoxonase and arylesterase activities have been extensively studied, whereas there is a lack of published evidence regarding the PON1 native lactonase activity (Dardiotis et al., 2019).

The association between PON1 and cardiovascular disease (CVD) so far has been investigated using several methods: 1. single nucleotide polymorphisms (SNPs) genotyping, 2. enzyme concentration measurement in biological specimens, and 3. PON1 enzyme activity measurement using different artificial substrates.

### Paraoxonase 1 gene polymorphisms and cardiovascular disease

Numerous clinical studies have shown association between different PON1 polymorphisms and variety of cardiovascular pathologies in the last two decades. Leus et al. were among the first to show that subjects carrying the homozygous LL/QQ genotype presented the biggest average carotid intima-media wall thickness compared to the other genotypes, suggesting it as a significant risk factor for carotid atherosclerosis in hyperlipidemic subjects (Leus et al., 2000).

Gnasso et al. investigated the association between HDL and the PON1-192 polymorphism in 347 subjects without CVD. Arg/Arg or Gln/Arg subjects reported a higher incidence of carotid abnormalities than Gln/Gln subjects, with a 3.27 OR (1.61–6.64 95% CI, *p* = 0.001) for a higher carotid score. Interestingly, it was seen that subjects with low HDL levels did not show an association between the PON1-192 polymorphism and carotid atherosclerosis. On the other side, subjects with higher HDL levels presented a significant correlation between the arginine-192 mutation and the occurrence of carotid atherosclerosis (Gnasso et al., 2002).

Gaidukov et al. showed that the 192 position acts in the binding of HDL. PON1-192Q presented with a three times lesser HDL affinity than PON1-192R, resulting in decreased stability, lactonase action and reverse cholesterol transport (Gaidukov et al., 2006).

Tsuzura et al. studied the correlation between plasma oxidized-LDL levels and PON1 in type 2 diabetes patients. The TT genotype of the -108C/T polymorphism led to an increased oxidized-LDL/apoB ratio, compared to the other genotypes (CT + CC: 0.55+/-0.11 vs. TT: 0.60+/-0.15, *p* = 0.02). A multiple regression analysis showed that the -108C/T polymorphism is the most significant nominator of the oxidized-LDL levels after apoB ones (Tsuzura et al., 2004). The TT genotype has been associated with lower HDL levels as well (Campo et al., 2004).

Regieli et al. led a 10-year study to follow 793 coronary artery disease (CAD) patients as part of the REGRESS (REgression GRowth Evaluation Statin Study) cohort, obtaining two PON-1 isotype genotypes (Q192R, rs662 and L55M, rs854560). The PON1-192Q and the PON1-55M isotypes were correlated with a bigger ischemic heart disease mortality risk, with an OR of 1.71 (1.0–2.8 95% CI, *p* = 0.03), and an OR of 1.56 (1.1–2.3 95% CI, *p* = 0.03), respectively. Both findings suggest a role of the PON-1 mutations in the 10-years risk of fatal CAD complications (Regieli et al., 2009).

The EPIC-Norfolk Prospective Population Study, conducted by Birjmohun et al. demonstrated a modest and inverse relationship between PON1-activity and risk of future CAD among 1138 apparently healthy men and women, over the 6-years follow up period. Even though the PON1-Q192R polymorphism showed to affect PON1-activity, it failed to associate with CAD risk (OR of 0.98, 0.84–1.15 95% CI, *p* = 0.8). PON1-Q192R was associated with the level of HDL-C (*p* < 0.0001) (Birjmohun et al., 2009).

de Souza et al. studied the correlation between the Q192R (rs662) and L55M (rs854560) polymorphisms and statin responses in 433 dyslipidemic subjects who used statin lipid-lowering therapy. Although the studied polymorphisms were not correlated with baseline lipid levels, both RR (χ(2) *p* = 0.009) and LL (χ(2) *p* = 0.026) homozygotes were underrepresented among those subjects that achieved their respective lipid goals, meaning that both polymorphisms may lead to interindividual variation in statin treatment response (de Souza et al., 2015).

Grzegorzewska et al. analyzed PON1 polymorphisms and paraoxonase activity in relation to dyslipidemia and cardiovascular morbidity and mortality in uremic settings. rs854560 AT+TT (OR of 1.28, 1.01–1.63 95% CI, *p* = 0.040), rs854560 TT (OR of 1.48, 1.04–2.11 95% CI, *p* = 0.031) and rs662 AA+AG (OR of 1.76, 1.10–2.80 95% CI, *p* = 0.018) had an impact on the incidence of dyslipidemia, identified by a ratio of triglycerides (TG) and HDL-C above 3.8. The measured paraoxonase activity showed a positive association with the incidence of dyslipidemia as well (*p* = 0.008). The PON1-163T isotype led to a higher incidence of ischemic stroke (OR of 1.38, 1.02–1.85 95% CI, *p* = 0.034). The PON1-108 TT genotype was associated with cardiovascular (*p* = 0.025) and cardiac (*p* = 0.018) mortality. It is important to note that the reported significances were derived by a multiple regression analyses that accounted for all routinely followed clinical parameters (Grzegorzewska et al., 2021).

### Paraoxonase 1 enzyme activity and cardiovascular disease

Reduced PON1 activity has been associated with numerous cardiovascular abnormalities. Kiss et al. measured the paraoxonase and arylesterase activities of PON1 in 37 systemic lupus erythematosus subjects and 30 matched control subjects. They showed decreased PON1 activity in lupus subjects, which was associated with clinical atherothrombotic complications (*p* < 0.01). Normal levels of arylesterase activity were reported in both study groups (Kiss et al., 2007).

Mackness et al. studied the effects of the G-909C and C-108T PON1 polymorphisms on the levels and enzyme activity of PON1, as well as on the incidence of coronary artery disease in 417 patients and 282 controls. Unlike the control group, coronary heart disease patients showed lower PON1 levels and paraoxonase activity, independently to the studied polymorphism genotype (*p* < 0.001) (Mackness et al., 2013).

Kunutsor et al. studied the link between PON1 and cardiovascular risk. They measured the arylesterase activity of PON1 in 6902 subjects. The subjects were then followed for approximately 9 years, during which they reported around 700 unwanted cardiovascular events. The circulatory paraoxonase activity was associated with multiple predictors of cardiovascular risk, such as HDL-C (*p* < 0.001), with a log-linear correlation with cardiovascular disease risk (Kunutsor et al., 2016).

Wysocka et al. investigated patients with confirmed CVD by measuring both paraoxonase and arylesterase PON1 activity towards paraoxon and phenyl acetate, respectively. They reported significant associations of paraoxonase activity and diabetes mellitus (*p* = 0.026) and premature CVD (*p* = 0.013). The arylesterase activity demonstrated positive association with the levels of HDL-C (*p* = 0.032) (Wysocka et al., 2019).

Murillo-Gonzalez et al. investigated the activities of PON1 related to arylesterase, paraoxonase, and lactonase, as well as different polymorphisms of PON1 (Q192R, L55M, T-108C and A-162G) in CVD patients. The CC- 108 genotype was significantly correlated with the incidence of cardiovascular disease, while all three PON1 activities were reduced in the disease group. The lactonase activity was shown to be a superior CVD marker (OR of 0.52), as opposed to the arylesterase activity (OR of 0.82) and the paraoxonase activity (OR of 0.99). A regression analysis failed to show an association between the PON1 lactonase activity and the studied polymorphisms (Murillo-González et al., 2020).

## Discussion

PON1 is able to reduce oxidized lipids, thus detoxifying oxidized-LDL, and degrade homocysteine thiolactone, a substrate that induces N-homocysteinylation of proteins. Vast scientific evidence has postulated PON1 as a beneficial factor in the setting of cardiovascular disease. Even though there are three identified PON genes in humans (PON1, PON2 and PON3), PON1 has received the biggest scientific interest (Chistiakov et al., 2017).

The liver is the primary expression site of PON1, which then releases it into the circulation where PON1 binds to the HDL particles. Given the fact that lactonase is the principal PON1 activity, polyunsaturated fatty acids that convert into lactone-like structures after oxidation are proposed as potential PON1 substrates. PON1 was shown to hydrolyze up to 19% of the lipid peroxides and 90% of the cholesteryl hydroperoxides in oxidized-LDL particles, suggesting a key atheroprotective action by peroxide LDL clearance (Chistiakov et al., 2017). Another possible natural substrate for PON1 is homocysteine thiolactone, which is known to induce atherogenic damage of endothelial cells by homocysteinylation of their structural proteins. PON1 detoxifies thiolactone by its hydrolysis to homocysteine (Jakubowski, 2010). PON1 also reduces oxidative stress induced by hydrogen peroxide, which makes PON1 an important anti-oxidative and anti-inflammatory agent (Chistiakov et al., 2017).

PON1 is one of the most investigated genes regarding predisposition to atherosclerosis based cardiovascular abnormalities. The two best-studied SNPs in the PON1 coding region, Met/Leu55 and Arg/Gln192, have both shown independent associations with the PON1 enzyme activity and lead to wide inter-/intra-individual variability (Chistiakov et al., 2017). Carriers of the 55Met/Met/-192Gln/Gln PON1 genotype have been characterized with the biggest anti-LDL oxidization potential, with strongest protective effects observed for the HDL-bound form of the enzyme (Bayrak et al., 2016). SNPs in the promoting PON1 gene region have been less extensively studied regarding their association with CVD.

When attempts were made to establish a causal role between PON1 polymorphisms and enzyme activity and cardiovascular risk, several meta-analysis gave conflicting conclusions.

Wang et al. reported a weak correlation of the Arg192 low-activity allele with higher CAD risk (OR of 1.11) in a meta-analysis of 88 case studies. When the analysis was done by only including studies that reported more than 500 CAD cases, the obtained correlation was further weakened (OR of 0.96), pointing to a possible publication bias in smaller studies published results (Wang et al., 2011). Tang et al. measured arylesterase and paraoxonase activities in patients without confirmed acute coronary syndrome scheduled for elective coronary angiography. Patients were followed for a period of 3 years for the appearance of myocardial infarction, stroke or death (MACE). Decreased levels of both paraoxonase and arylesterase activities were shown to be correlated with augmented MACE risk. Surprisingly, the arylesterase activity appeared to be a better prognostic marker (*p* < 0.01), even after the addition of traditional cardiovascular risk factors (*p* < 0.01), in both primary and secondary MACE prevention. As opposite, an association study including nearly 80 000 subjects reported various PON1 polymorphisms to be correlated with both arylesterase and paraoxonase PON1 activities, but no correlation was reported between the PON1 polymorphisms and a history of cardiovascular disease or the 3 year risk of MACE (Tang et al., 2012). The meta-analysis done by Kunutsor et al., including around 15 000 subjects and 3000 reported CVD outcomes, did not show an improvement in the CVD risk classification after the addition of the PON-1 levels in the used risk model, with an adjusted relative CVD risk of 0.95 (*p* = 0.138) per one SD elevation in PON-1 levels (Kunutsor et al., 2016).

A recent study by Sikora et al. showed proatherogenic plasma proteomes shifts as a result of genetic PON1 level depletion. The PON1-Q192R polymorphism was shown to affect mainly proteins involved in the lipoprotein metabolism, such as upregulation of APOM and APOD in subjects carrying the PON1-192QR genotype, apolipoproteins that inhibit lipid oxidation, downregulation of APOC1 and APOA1, apolipoproteins that regulate cholesterol transport, and upregulation of APOB, an apolipoprotein that stimulates cholesterol retention. Both findings suggest a proatherogenic proteomic shift by reducing PON1 levels. The PON1- 192QQ genotype was associated with downregulation of F13B and SERPINA1, both involved in the coagulation process, thus increasing the risk of atherothrombosis (Sikora et al., 2020). Serum PON1 concentrations in the general population show very high variability. This phenomenon has postulated a debate the PON1 phenotype or the PON1 genotype, meaning weather the qualitative or the quantitative aspect of the enzyme, is primarily correlated with CVD risk. What can be seen clearly is that every factor that influences PON1 serum levels will certainly influence the HDL anti-oxidative potential, thus be associated with atherosclerosis.

## Conclusion

Given the previously postulated, we can deduce that carriers of PON1 polymorphisms known to lead to augmented enzyme levels and activity, may have lower susceptibility to oxidation and inflammation, thus lowering the incidence of accelerated atherosclerosis and CVD, and vice versa. Therefore, in patients with high and very high cardiovascular risk, in whom there is no deviation in the quantitative level of lipid fractions, it seems reasonable to perform PON1 genotyping in order to prove the existence of PON1 polymorphisms associated with reduced HDL protection.
